# Using SNSs for early detection of disease outbreak in developing countries: evidence from COVID-19 pandemic in Nigeria

**DOI:** 10.1016/j.heliyon.2021.e07184

**Published:** 2021-05-31

**Authors:** Tunde Adebisi, Ayooluwa Aregbesola, Festus Asamu, Ogadimma Arisukwu, Eyitayo Oyeyipo

**Affiliations:** a SDG 16, Peace, Justice and Strong Institutions, SDG 10, Reduced Inequality, Department of Sociology, Landmark University, Omu-Aran Kwara, Nigeria; b SDG 10, Reduced Inequality, Department of Sociology, Landmark University, Omu-Aran Kwara, Nigeria; c SDG 4, Quality Education, Centre for Learning Resources, Landmark University, Omu-Aran Kwara, Nigeria

**Keywords:** Public health surveillance, COVID-19, Twitter, Nigeria, Qualitative research

## Abstract

Developing countries, particularly Nigeria, continually find it challenging to proactively and actively carry out early-stage surveillance for disease outbreaks due to the lack of quality workforce, a dearth of public health data, and the absence of automated surveillance systems in the country. This study presents the potential and ability of Twitter in tracking early detection of COVID-19, monitoring the dissemination of information, and exploration of public awareness and attitudes among Nigerians. Tweets mentioning COVID-19 and related keywords were collected in 11 batches via the NCapture™ plugin available on Google Chrome from February 20 - May 6, 2020. The analysis includes a time series analysis to track the distribution of data and content analysis to analyze the knowledge and attitudes of Nigerians. A total of 67,989 tweets (1,484 unique and 66,505 retweets) citing COVID-19 and related keywords were returned. The Tweets started to emerge earlier to the first confirmed case in Nigeria while maintaining a dangling-upward movement up to the 11th week under study. Matters arising from the tweets include a dearth of information on COVID-19 and optimism among others. The results provide insight into the intersection of SNSs and public health surveillance. Results show how helpful Twitter is to educate education in public health. Health organizations and the government may benefit from paying attention to both amusing and emotional contents from the Twitter community to formulate a viable policy for treatment and control.

## 1 Introduction

Coronavirus disease (COVID-19) was initially called Novel Coronavirus (2019-nCoV) during its earliest stages of discovery in Wuhan City, Hubei Province of China (WHO, 2020d; Cleland et al., 2020). The disease was announced by the World Health Organization (WHO) as a pandemic on March 11, 2020 (Cleland et al., 2020; WHO, 2020b); the first pandemic since the H_1_N_1_ swine flu in July 2009 (WHO, 2009). Nigeria confirmed her first case of COVID-19 in Lagos State on February 27, 2020, when an Italian who works in Nigeria returned from Milan, Italy to Lagos, Nigeria on the 25th of February 2020 (Nigeria Centre for Disease Control, 2020a, b). As of May 4, 2020, more than 3, 435, 894 people globally have been infected with the virus, out of which 239, 604 persons had died with other several active cases. The reported number of infected people in Africa was 30, 536, and 2, 388 from Nigeria, with 1,085 and 85 deaths respectively according to the World Health Organization (WHO), although the Nigeria Centre for Disease and Control (NCDC) reported a total of 3,047 cases in the country (WHO, 2020a; Nigeria Centre for Disease Control, 2020a).

During the earliest period of the COVID-19 pandemic, a combination of practices was imitated by many countries to contain the pandemic, reduce the death toll, and assuage the climate of fear. Many countries, including Nigeria, introduced multi-level response strategies like; lockdown, contact tracing, and self-isolation or quarantine; public health procedures such as hand washing and/or sanitizing, social distancing, and the use of face mask; anticipating severe health cases that could require isolation, oxygen, and mechanical ventilation; prevention of and management of infections in healthcare facilities; and delaying or canceling large-scale public meetings (Bedford et al., 2020; Sjödin et al., 2020). However, despite various interventions, particularly in Nigeria, surveillance, and containment remains difficult, not just because of the adverse effect of a typical infectious pandemic disease (WHO Regional Office for Africa, 2016), but also as a result of poor surveillance of public health outbreaks in the country (WHO, 2001; Nnebue et al., 2014; Isere et al., 2015; Idiegbeyan ose et al., 2020; Luret et al., 2015; Fall et al., 2019). With COVID-19 in Nigeria and the upsurge in the daily number of reported cases across the country (Nigeria Centre for Disease Control, 2020a), public panic is expected to be on the rise as a result of fear. On the other hand, it is also possible for the public to experience what is referred to as “caution-fatigue”; a situation where individuals become impatient with warnings, don't believe the warnings to be relevant or de-emphasize the actual risk (Gollan, 2020). Consequently, the certitude of containing the disease in the nearest future is unknown.

Surveillance of public health involves monitoring of disease incidence and events related to health to enable timely intervention in disease control (Buehler et al., 2004). Globally, health surveillance systems are playing a major role in both outbreak detection and response management of Infectious Diseases (IDs). However, in developing countries, disease outbreaks are difficult to monitor due to some reasons. Quality of manpower, scarcity of public health data, and absence of automated surveillance systems have all been identified (Lur et al., 2015). Currently in Nigeria, the collection, collation, analysis, and interpretation of disease-related data in public health institutions are often incomplete and untimely (Abdulraheem et al., 2004; Luret et al., 2015; Fall et al., 2019. For improved surveillance systems, a range of priority steps is recommended. The use of digital technology to promote inter-agency contact with the public. WHO had developed interim global surveillance for COVID-19, which allows ‘Case-based reporting’, ‘Aggregated reporting, and ‘Member State Self-Reporting Platform by the national authorities of WHO member states (WHO, 2020c). Similarly, the Federal Ministry of Health (FMoH) had before now, developed a conceptual framework for preparedness and response to public health concerns at both epidemic and pandemic levels. The framework was modeled on the WHO ‘Pandemic Alert Phasing Protocols’ as well as on various stages of the 'pandemic curve’(Federal Ministry of Health, 2013). Surveillance and Laboratory was the first strategy enlisted by FMoH, should the country experience a pandemic.

The earliest phase of the COVID-19 pandemic in Nigeria can be exemplified by the earliest lockdown phase in the country. Nigeria first went into lockdown in late March following the first case on February 28 and the successively increased cases of coronavirus in the country. However, on April 27, President Buhari approved a phased and gradual easing of lockdown measures in three states; Abuja, Lagos, and Ogun. The total lockdown was later reduced to a curfew from 5 pm till 6 am beginning from 4 May, although some states modified the curfew hours to commence at 10 pm and expire at 5 am (Al Jazeera, 2020a; Al Jazeera, 2020b).

In an early phase of a pandemic like the 2019-nCoV, defined as ‘*WHO Phase 4: Nigeria Sub-Phasing: Phase 4c*’ ‘Surveillance and Laboratory’ action should include; providing pandemic surveillance advisories to the health community; underscoring the necessity for correct and prompt reporting to surveillance cohorts; improve surveillance to detect early cases, evaluate viral virulence and ascertain exclusive viral physiognomies; improve laboratory action protocols to intensify capacity at significant laboratories; Observe the health alert networks and extra sources of pandemic statistics; surveillance for animal disease occurrences that are possible to impend the human populace; Deliver intermittent updates to significant leaders, home and foreign organizations and other significant stakeholders; Confirm that data reported to WHO follows the International Health Regulations (IHR). The overall surveillance data will aid government response to the pandemic (Federal Ministry of Health, 2013). ‘Surveillance and Laboratory’ action in Nigeria remained almost the same despite that the pandemic is entering a different and more severe phase (Nigeria Pandemic Alert Phases 4f, 5f and 6f), where impacts are severe on the health sector and potentially severe on non-health systems such as; the disruption of utility, transportation, public safety, and practically every other aspect of economic and social infrastructure (Federal Ministry of Health, 2013; Osayantin et al., 2020; IseOlorunkanmi et al., 2021).

While emerging diseases should be viewed with national surveillance and control strategies, resources are often limited (Fall et al., 2019), especially in Nigeria (Ali et al., 2016). Since surveillance systems are not timely, accurate, effective, or adaptable, information gaps can occur (Hall et al., 2012; Abdulraheem et al., 2004). Optimal public health outbreak surveillance should, therefore, use multiple data collection, analysis, and dissemination techniques in an exhaustive way (Khan et al., 2010). Tools for the transfer of knowledge to support the management of outbreaks are therefore needed (Ridley, 2004; Kieny et al., 2016). Social Network Sites (SNSs) allow the public to play an active role in news event coverage and diffusion. Users express their perspectives, thoughts, and fears when transmitting health incidents beyond the context of public health (Khan et al., 2010; Sullivan et al., 2012; Ahmed et al., 2010).

The need to create and exchange knowledge related to health is increasingly relevant for tracking outbreaks (Odlum and Yoon, 2015). The literature already published papers about how educators reacted to or may respond to COVID 19, along with several comments and letters explaining how it has either changed or would change due to COVID-19. However, a remarkable feature of the COVID-19 response involves the crucial significance of public health, its use of data, and modeling (Ellaway et al., 2020). Nigeria's national response to the COVID-19 pandemic has sustained reliance on science, data, and experiences drawn from other nations, and consideration of the country's peculiar environment to address the pandemic while maintaining the guidelines issued by WHO (OSGF, 2020). Surveillance via electronic media, on the Internet, offers significant opportunities for public health practice (Khan et al., 2010). According to Signorini et al. (2011), one of the most common SNSs is Twitter, a microblogging platform that allows tweeting (reporting, sharing, and addressing news events that can provide valuable information) (Mittal and Patidar, 2019; Bae and Lee, 2012).

Twitter users communicate via direct messages or implored answers, which can be disseminated primarily via retweeting (forwarding) (Odlum and Yoon, 2015). In Nigeria, Twitter accounts for the largest SNSs after Facebook with 30.4% of the entire social media users. Its usage increased by 6.33% since the first case of the Coronavirus in February 2020 (GlobalStats, 2020). Twitter has been seen as an evolving broadcasting platform for public health education and news, demonstrated by its utility during H_1_N_1_ pandemic planning activities in 2009 and the Ebola Virus Disease (EVD) outbreak in 2014 (Sullivan et al., 2012; Odlum and Yoon, 2015). The majority of its users are youths who have been characterized as very innovative and constantly try out new things, approaches, or methods in any activity they get involved in (Adebisi et al., 2020). The ability of Twitter in terms of broad reach, timeliness, and low overhead captures prevalent disease trends, collects information, and disseminates knowledge. More so, its usefulness supports its ability in new and creative ways to affect public health outbreak surveillance (Khan et al., 2010; Odlum and Yoon, 2015).

This study aims to explore the use of an effective SNS tool during an emergency in public health and provide a snapshot of the pandemic COVID-19 tweets to capture early detection of disease, monitor the information trends, and assess public knowledge and attitudes. This is born out of the belief that the steps to manage COVID-19 should be considered a socio-material procedure (Cleland et al., 2020). Twitter has proved to be effective in public health surveillance particularly during the Ebola outbreak in West Africa (Odlum and Yoon, 2015; Vorovchenko, 2015). When compared with public health workers and experts, the Twitter community may be less knowledgeable about the dynamics of a particular disease, however, their tweets usually coincide with news events and express their attitudes towards such disease. Tweets on such outbreaks can therefore provide a unique opportunity for public health organizations and the government to listen to their audience/citizens to share scientifically accurate information, and formulate a viable policy for treatment and control.

## 2 Methods

This research draws from Odlum and Yoon (2015) and its method was approved by Landmark University Centre for Research, Innovation, and Development (LUCRID).

### 2.1 Data collection

This study collected tweets (unique and retweets) in batches via the Google Chrome-based version of NCapture™; a free web-browser extension that allows researchers to quickly and easily capture content like web pages, online PDFs, Twitter tweets, and Facebook posts for import into the NVIVO software, (a qualitative data analysis computer software that helps qualitative researchers to organize, analyze and find insights in unstructured or qualitative data like interviews, open-ended survey responses, journal articles, social media, and web content, where deep levels of analysis on small or large volumes of data are required).

### 2.2 Data sampling

Data captured tweets from February 20, 2020; a week before February 27, the date of COVID-19 first case in Nigeria; and May 6, 2020; two days after the commencement of lockdown relaxation in Nigeria. This is done to understand the information dissemination trends, public perception, and attitudes to the COVID-19 pandemic particularly at the early stage of the pandemic. Each batch of tweets consists of 7 days tweets starting from February 20 through May 6, 2020, totaling 11 weeks.

The tweets are collected in English and the keywords used for the identification of COVID-19 related tweets in Nigeria were informed by trending words on Twitter and most search words on Google between February 20, 2020, and May 6, 2020. They include; coronavirus, COVID-19, COVID, staysafe, lockdown, distancing, curfew, quarantine, pandemic, and palliatives. Through the advanced search option available on Twitter, all keywords generated a dataset within a specified time range were collected. Elements of the data collected for every tweet included timestamp, username, content (unique tweet), and retweet(s).

### 2.3 Data analysis

To assess COVID-19 information dissemination trends, the number of posts (tweets and retweets) were categorized by dates within the week before the first case of COVID-19, and the commencement of lockdown relaxation in Nigeria. Using Microsoft Excel Charts, the descriptive statistics, including the bulk of posts (tweets and retweets) within the country were linked with the segmented time range. Also, a content study (Arisukwu et al., 2021) was carried out using the NVIVO software to capture natural language processing to collect public information, expectations, and attitudes about COVID-19.

## 3 Results

### 3.1 Trends of information spread with time

**Figure 1:**
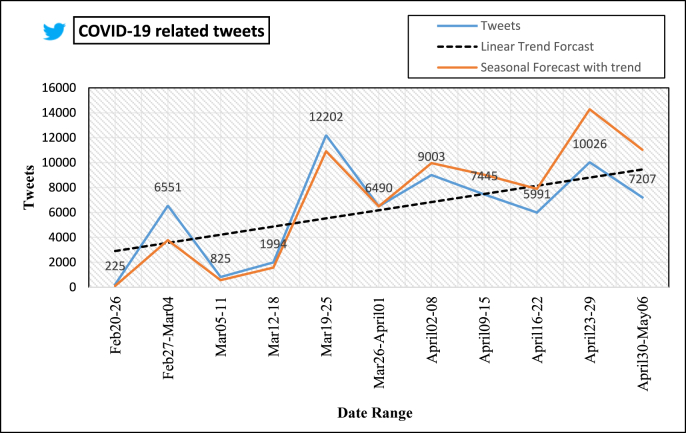
Trend of Information spread and time trends in COVID-19 related tweets between a week before the first reported case and the end of the first phase of National lockdown in Nigeria. NCDC.

**Figure 2:**
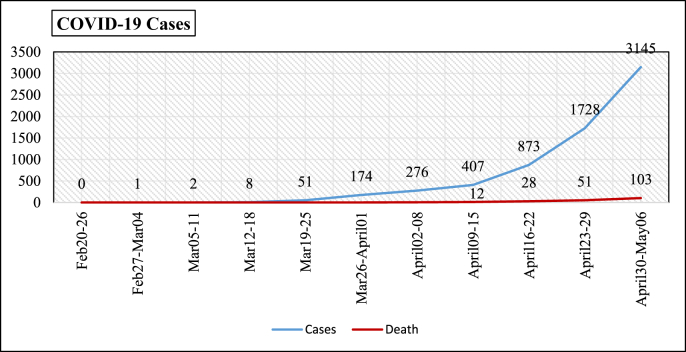
COVID-19 Cases between a week before the first reported case and the end of the first phase of National lockdown in Nigeria. NCDC.

In Nigeria, a sum of 67,989 tweets citing COVID-19 and related keywords were returned (1,484 unique and 66,505 retweets) from February 20 - May 06, 2020. Exactly a week before the first reported case in Nigeria, a total of 225 tweets (0.4%) were returned. It represented the lowest number of tweets across the 11 weeks under study. Following the announcement of the first case in Nigeria, Twitter experienced an upsurge in the number of tweets mentioning coronavirus and related keywords for the next week; February 27 – March 04, 2020. A total number of 6,551 tweets were captured. For the third week; March 05–11, 2020, there was an outrageous decline in the curated tweets. Only 825 tweets were mentioned. The number of tweets then begin to rise again for the fourth week (1,994 tweets) and fifth week (12,202 tweets); the highest number of tweets recorded during that period. The sixth week; March 26 – April 01, experience another notable decline in the number of tweets (6,490 tweets). It however slightly increase in the seventh week (9,003 tweets) and slightly decline in both week eight (7445 tweets) and week nine (5991 tweets). Week ten saw a massive increase only second to week five (10,026 tweets) and then decreases on week eleven (7,207 tweets). Arriving at the linear trend forecast, the intercept of the spread was calculated as 2250.22, while the slope is at 654.65 (See Figures 1 and 2).

**Figure 3:**
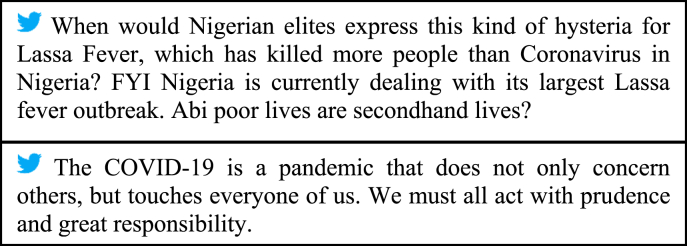
Perceptions and attitudinal change that COVID-19 belongs to the rich only.

**Figure 4:**
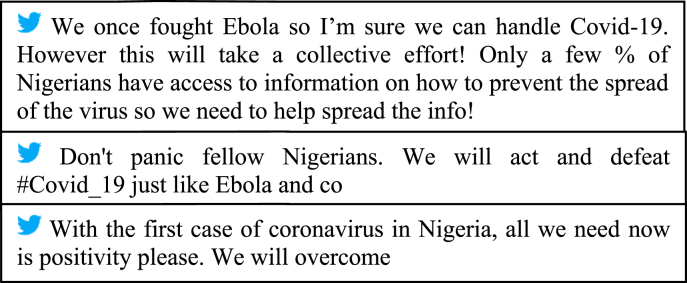
Expression of optimism.

**Figure 5:**
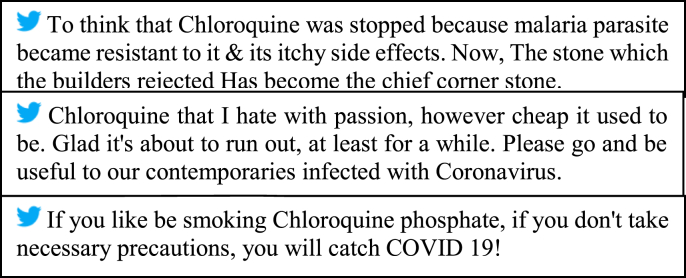
Chloroquine, as a potential cure for COVID-19 pandemic.

**Figure 6:**
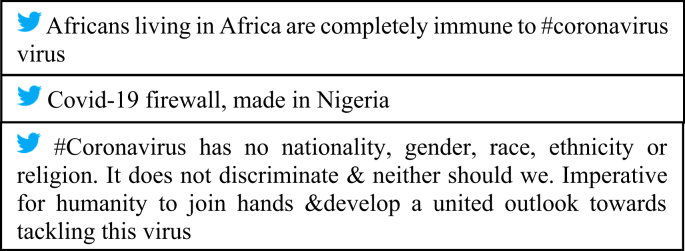
Rumor that ‘black blood’ was immune to the virus.

**Figure 7:**
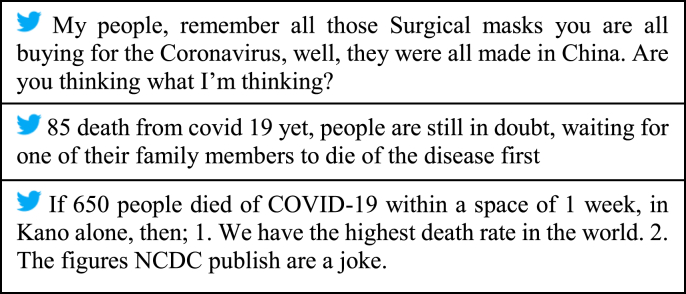
Discussion around the fact COVID-19 is an imported virus (Index case).

**Figure 8:**
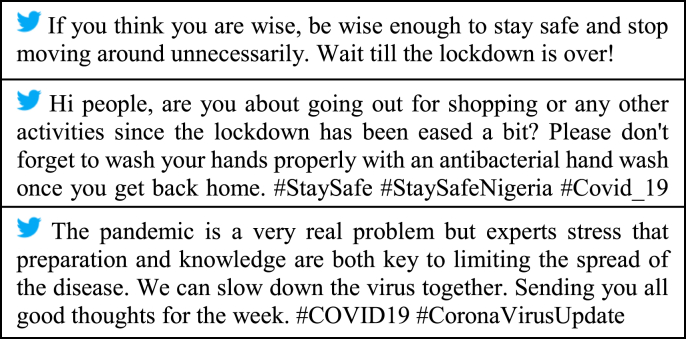
Discussions following heightened community spread and death, people seemed to take the virus more seriously.

**Figure 9:**
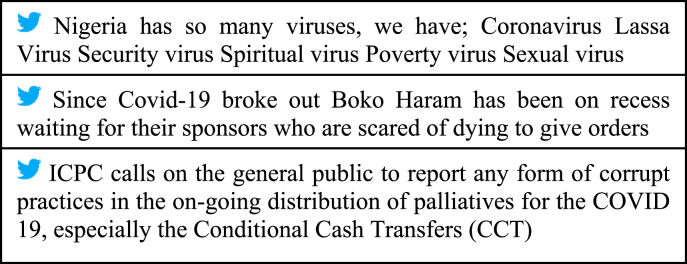
Narratives about various other issues that are as sacrosanct and deadly as COVID-19; other diseases, poverty, killings and corruption.

**Figure 10:**
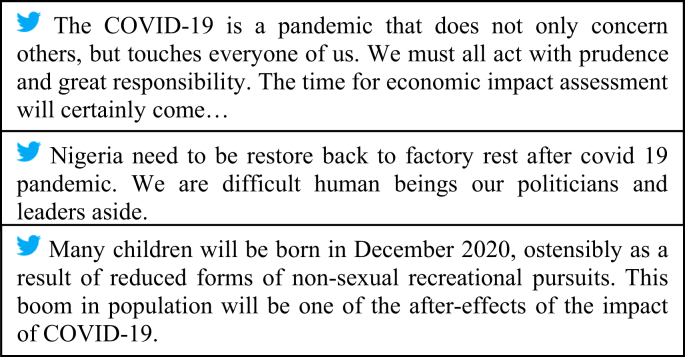
Some economic, social, and political concerns; the consequence of the pandemic and its curtailing measures. Some of the good that a platform like Twitter has done include rapid novel information dissemination; conventional platforms like textbooks and journals do not have this type of reach.

#### 3.1.1 Early pandemic detection

Tweets mentioning coronavirus and related keywords started to emerge on Twitter earlier to the official alert of the first confirmed case. Twitter community in Nigeria discussed several subjects around the pandemic. At this time, Nigerians on Twitter use the coronavirus and other trending hashtags majorly to promote or disseminate their tweets to a wider audience even when the tweets have nothing to do with the COVID-19. For instance, in the tweet below:

“Relaxing. Unwinding. Chilling. The Xovar life goes by many names. Winking face Face with tears of joy Start your week with a wide smile! ∗∗∗ #MondayMotivaton #mondaythoughts #lagos #nigeria #UnilagBlackOut #Covid_19 #HelloDearEx”).”

After the first confirmed case of coronavirus in Nigeria, coronavirus-related tweets increased. Tweet frequency steadily increased and declined with reported cases of coronavirus in Nigeria from February 20 – March 18, 2020. The number of reported cases has been steadily increasing up to only 8 confirmed cases in the country. Twitter, however, exploded with 12,202 tweets (unique and retweets) between March 19 and March 25 as official sources in the country had reported 51 confirmed cases in the country. Nigerians must have been bewildered by the outrageous number of cases in the country as series of tweets expressed fear, and confusion. A tweet with 2,700 retweets explain the upsurge of confirmed cases:

(“Let me explain this Coronavirus thing in network marketing Each infected person with COVID19 is expected to bring two new people in, those two new people are expected to bring 4 people in, and so on. Do the math”).

### 3.2 Content analysis

The tweets show so many dynamics in the pattern of thought of those posts. From the data set. The following assumptions are deducible:

#### 3.2.1 Perceptions and attitudinal change that COVID-19

In the early stages of the virus (20^th^ February to mid-March), some of the popular perceptions of the various included that it is an elitist virus only common to the rich. Moreover, as the infection rate increased and fatality worsens, narratives began to change (See Figure 3).

#### 3.2.2 Expression of optimism

Nigerians expressed optimism in overcoming the virus if the information is adequately disseminated (See Figure 4).

#### 3.2.3 Chloroquine, as a potential cure for COVID-19 pandemic

Chloroquine was seen as a potential cure for the COVID-19 pandemic (See Figure 5).

#### 3.2.4 Rumour that ‘black blood’ was immune to the virus

It was also rumour that ‘black blood’ was immune to the virus (See Figure 6).

#### 3.2.5 Imported virus

Discussion around the fact that it is an imported virus (Index case) was also quite popular. The change of attitude mentioned in the previous point was also aided by increased community spread of the virus in Nigeria (See Figure 7).

#### 3.2.6 Transistions in narratives following the heightened community spread and death

From Mid-March to late April (following heightened community spread and death) people seemed to take the virus more seriously. Various measures at curtailing the spread began to trend more and more honest conversations surfaced. Tweets about measures like lockdown, social distancing, and washing of hands became very trendy. Knowledge was recognized as power and fundamental in defeating the virus (See Figure 8).

#### 3.2.7 Other problems that needs urgent attention in the country

Alongside this new narrative about the various are other issues some of these sTwitter users believe are sacrosanct and deadly- Lassa fever, Poverty Virus, Killings across different parts of the country. With lockdown and government intervening measures came various challenges like corruption-distribution of palliatives, ventilator crisis, and the poor state of healthcare facilities across the country, etc (See Figure 9).

#### 3.2.8 Some economic, social and political concerns

Following these concerns are the economic, social and political consequence of the pandemic and its curtailing measures (See Figure 10).

## 4 Discussion

Valuable and accurate knowledge is the basis for monitoring disease outbreaks (Hall et al., 2012). This study shows how tweets can be collected and analyzed to assist early warning systems for disease outbreaks and to track health education messages (Tappero and Tauxe, 2011). Besides, the Centre for Disease Control (CDC) has proposed that public health education crises involve researching public awareness and perceptions and disseminating mass media communications. Twitter, offers the capacity to promote the attainment of these objectives that offer additional opportunities of improving health education in respect to individual, period, and location (Hall et al., 2012).

### 4.1 Timely detection and early signals

This study shows how Twitter promotes and aids early warning systems for the monitoring of disease outbreaks. The specific dataset represents the early phase of public health notifications about the recent COVID-19 outbreak in Nigeria. This study has two novel findings. The result showed a dangling-upward movement in the number of tweets citing COVID-19 and related keywords beginning on February 20, 2020, a week before the official reported case in Nigeria. Although Twitter is adopted and used in resource-limited places like Nigeria, it has been shown that the number of related COVID-19 tweets increased during the days leading up to the official news alerts. The results show how Twitter is used in areas where monitoring systems for disease outbreak monitoring are not optimal for promoting early warning systems. Prompt systematic collection, interpretation, and transmission during break-out and surveillance efforts of health-related data are essential for containment and control (Henning, 2004). Failure to acknowledge a threat to public health or lack of interventions can result when information is not available in good time (Hall et al., 2012). Africans have increased their participation in global discussions. There have been reports of social media development in Nigeria (GlobalStats, 2020) and Africa (Odlum and Yoon 2015). Studies have shown Africans to use SNSs like Facebook and Twitter primarily on their cell phones online (Odlum and Yoon, 2015). Nothing may ignore or exceed the SNS value. SNS data are and will continue to be useful in support of global health programs and findings (Essoungou, 2010).

### 4.2 Nigerian perceptions, needs, and education

Nigerian perceptions have been analyzed via the trend of information spread with time. The communication revealed and news alerts have been reflected by public concern. Tweets occurred in connection with health warnings, precautions, solidarity, and distrust in government ability, similar diseases, and the aftermath of the pandemic. It was noted that the frequency and fear of COVID-19 related tweets have a dangling-upward movement, with rising numbers of cases and deaths in the country. Although tweeting increased across the country, the highest number of tweets in the selected time range was captured between March 19 and March 25 as official sources reported 51 confirmed cases in the country.

Dissemination of health information is important during disease outbreak response in support of public health interventions (Odlum and Yoon, 2015). However, to ensure effective communication, the need for health education must be effectively disseminated. Results from this study showed how the analysis of tweets can be valuable to identify and measure the necessity to intervene in public health. Messages are spread and widely spread by tweeting. Results show that health alerts are effectively disseminated. On February 27 (the date of COVID-19 first case in Nigeria) the number of retweets in the COVID-19 public concern increased significantly. Generally speaking, tweets looked for health information and confirmed that appropriate messages were needed to accompany health education warnings. It can be contended that prevention messages will be instantaneously insignificant following health warnings (Odlum and Yoon, 2015). Results from this study show that news of COVID-19 education messages was still minimal one week after the official report on February 27, 2020. In the early periods of news and health alerts few health organizations and individuals tweeted about COVID-19 prevention. However, due to limited follow-up and reach, there was insufficient distribution and no major news agencies retweeted such prevention messages. The following institutions do not represent the general population and have higher functional and health literacy.

The COVID-19 tweets reflected several topics. Most themes that identified knowledge lacunae included deficiencies in health education. COVID-19 information was both provided and requested. Users of the Twitter SNSs requested information on infection, preventative activities, and the government's ability in containing the outbreak. This knowledge offers an insight into particular areas for mass delivery of health education. Generally, the positive and negative impact of Twitter as a medium of information amidst a major pandemic like COVID-19 must be examined. In doing this, it will be important to also state the role Twitter is playing in censoring potentially harmful information (infodemics). Some of the impacts of this harmful information that trended this period can cause severe mental health issues; spread of misinformation. To counter rumors and claims from social media products aimed at preventing COVID-19, Twitter was used by NCDC to provide a warning (NCDC, 2020). Some of the good that a platform like Twitter has done include rapid novel information dissemination (conventional platforms like textbooks and journals do not have this type of reach).

Compassion, in the form of motivation, was also identified in the analysis as Twitter users spread the message of hope and optimism. Results further support the need to improve educational efforts to diminish fear as evidenced in many of the captured tweets. Given that the spread and death toll of COVID-19 in Nigeria has increased rising fear is unavoidable. Displaced anxiety leading to irrational thinking or actions is a significant obstacle to the prevention, control, and treatment of outbreaks.

## 5 Conclusions

To complement news warnings on COVID-19 and pandemics, there is a current, urgent need for successful health education communications. In recent COVID-19 cases in Nigeria, an official report, particularly NCDC released many confused and ineffective communications, bringing legislation into question the capacity of the department to deal with the COVID-19 health crisis (Nigeria Centre for Disease Control, 2020b). While awareness is available, Nigerians remain confused, frightened, and unbelieving. In the alternative, during the Ebola Disease Virus (EDV) outbreak in Nigeria, the influence of health education and communication in Nigeria is something to be experienced. The WHO had praised the country for their swift and effective public health response by rapid monitoring and isolation as an epidemiological implementation of the international standing (Odlum and Yoon, 2015). The successful response of Nigeria during the EDV outbreak confirms the importance of health communication. Similar mass distribution activities can be assisted by Twitter.

Twitter is immense in outbreak monitoring that allows data to be captured in real-time. SNSs are increasingly being used globally. A broader concept of outbreak monitoring in public health is important because the information in social media can be used to promote and improve existing early warning systems. Regardless of character restrictions, Twitter users give many dimensions of concern. Our analysis shows no determination in health education, despite obvious public unease. Twitter enables public participation and feedback by government and health departments during outbreak monitoring activities. Twitter will remind content of desired results for efficient data messaging. Entry to these data makes it possible to determine the behavioral response with greater precision and sensitivity. Since fear and awareness deficits fuel epidemics, population-specific and literacy-specific health education campaigns need to be supplemented by disease warnings to help deter and monitor action.

## Declarations

### Author contribution statement

Tunde Adebisi: Conceived and designed the experiments; Performed the experiments; Analyzed and interpreted the data; Wrote the paper.

Ayooluwa Aregbesola: Contributed reagents, materials, analysis tools or data.

Festus Asamu: Analyzed and interpreted the data.

Ogadimma Arisukwua: Contributed reagents, materials, analysis tools or data; Wrote the paper.

Eyitayo Oyeyipo: Performed the experiments; Wrote the paper.

### Funding statement

This research did not receive any specific grant from funding agencies in the public, commercial, or not-for-profit sectors.

### Data availability statement

Data associated with this study has been deposited at Mendely Data under the accession number https://doi.org/10.17632/kbhb7cpvfb.1.

### Declarations of interests statement

The authors declare no conflict of interest.

### Additional information

No additional information is available for this paper.
